# Survival and Tumour Microenvironment in Spatially Distinct Regions in Patients Resected for Hepatocellular Carcinoma: A Multicentre Study

**DOI:** 10.1007/s12029-026-01467-1

**Published:** 2026-04-15

**Authors:** Sophie Bull Nordkild, Jeanett Klubien, Delal Akdag, Colm O’Rourke, Jeanette Baehr Georgsen, Patricia Switten Nielsen, Torben Steiniche, Gerda Elisabeth Villadsen, Anders Riegels Knudsen, Gro Linno Willemoe, Jesper Bøje Andersen, Susanne Dam Nielsen, Hans-Christian Lykkegaard Pommergaard

**Affiliations:** 1 Department of Digestive Diseases, Transplantation and General Surgery, Rigshospitalet, Inge Lehmanns Vej 7, Copenhagen Ø, 2100 Denmark; 2 Hepatic Malignancy Surgical Research Unit (HEPSURU), Department of Digestive Diseases, Transplantation and General Surgery, Copenhagen University Hospital, Copenhagen, Denmark; 3 Viro-immunology Research Unit, Department of Infectious Diseases, Rigshospitalet, Copenhagen University Hospital, Copenhagen, Denmark; 4 Biotech Research and Innovation Centre (BRIC), Department of Health and Medical Sciences, University of Copenhagen, Copenhagen, Denmark; 5 Department of Pathology, Aarhus University Hospital, Aarhus, Denmark; 6 Department of Clinical Medicine, Aarhus University, Aarhus, Denmark; 7 Department of Hepatology and Gastroenterology, Aarhus University Hospital, Aarhus, Denmark; 8 Department of Surgery, Aarhus University Hospital, Aarhus, Denmark; 9 Department of Pathology, Rigshospitalet, Copenhagen University Hospital, Copenhagen, Denmark; 10 Institute for Clinical Medicine, Panum Institute, University of Copenhagen, Copenhagen, Denmark

**Keywords:** Hepatocellular carcinoma, Liver resection, Tumour microenvironment, Prognosis, Immunohistochemistry, Immune cells

## Abstract

**Introduction:**

The tumour microenvironment (TME) may play a pivotal role in the development, progression, and prognosis of HCC. We aimed to investigate the prognostic association of the TME in Danish liver-resected patients with HCC.

**Methods:**

We included all patients liver-resected for HCC between January 2000 and December 2016 with available tumour tissue stored in the diagnostic biobank. Resected tumour tissues and corresponding normal liver tissues were investigated for immune cell densities with immunohistochemistry. We investigated the association between individual immune cell densities and prognosis. We identified distinct patient groups based on hierarchical clustering and heatmap visualisation, for their association with prognosis.

**Results:**

We included 75 patients, 72% were male and median age was 66 years (58-72). Most patients had a single tumour, median size was 50 mm, and 57% without vascular invasion. Median follow-up was 44 months and 50% of the patients had a recurrence within the study period. The density of CD4+ T cells in the invasive margin was significantly associated with a higher risk of mortality (HR = 1.43, 95% CI: 1.13–1.82), whereas a higher density of tumour-associated macrophages in the tumour centre was significantly associated with a lower risk of cancer-related mortality (HR = 0.53, 95% CI: 0.28–1.00). Clustering of patients based on immune cell densities in tumour tissue revealed that those with lower immune cell counts exhibited significantly worse disease-free survival when compared to other patients (*p*= 0.03).

**Conclusion:**

The TME had a prognostic association with survival, lending credence to spatial immune cell densities as important factors contributing to prognosis.

**Supplementary Information:**

The online version contains supplementary material available at 10.1007/s12029-026-01467-1.

## Introduction

Hepatocellular carcinoma (HCC) is the sixth most prevalent cancer and the third leading cause of cancer-related death globally [1]. The primary causes include viral hepatitis, alcoholic steatohepatitis, and metabolic dysfunction-associated steatohepatitis, all of which trigger chronic liver inflammation, cirrhosis, and HCC [2]. Currently, curative treatment options for HCC include liver ablation, surgical resection, and liver transplantation [2–4]. However, the prognosis remains poor, largely attributed to late-stage diagnosis and a high prevalence of comorbidities. Moreover, despite curative treatment, recurrence rates are high [5]. Previous studies have implied that molecular features of the tumour and the liver environment may be among important factors for recurrence [6–8].

As HCC emerges, the composition of immune cells within the parenchyma and tumour shifts significantly, creating a complex tumour microenvironment (TME) distinct from a healthy liver [9, 10]. The TME is characterised by an abundance of immune-suppressive cells and a sparse presence of immune-supportive cells, due to exhaustion of the immune-supportive cells, a hindrance of the cells reaching the tumour, or an overpowering of the immune-suppressive cells. Thus, the TME plays a pivotal role in the proliferation, migration, invasion, and angiogenesis of HCC [11]. The TME of HCC is not consistently universal in its composition, however, the pro-tumour environment often contains a large number of regulatory T cells (Tregs), characterised by the expression of FOX-P3. As a main component of the natural immune response, Tregs maintain tolerance and prevent autoimmunity by hindering further activation of other immune cells using inhibitory receptors, such as programmed death-ligand 1 (PD-L1). Ultimately, in HCC, Tregs dampen the ability of the immune system to detect and respond properly to the progressing falters, proliferation of stimulatory immune cells is impaired, and the anti-tumour immune response, mediated by cytotoxic T cells (CD8 + T cells) and natural killer cells (NK cells), diminishes. Finally, tumour-associated macrophages (TAMs) and tumour-associated neutrophils (TANs) further amplify this immune-suppressive shift in the TME by activating additional Tregs, further dampening the anti-tumour immune response [12, 13]. Subsequently, tumour growth and progression are promoted, directly affecting the prognosis of HCC [13, 12, 14–17].

Extensive research has been performed on the TME of many cancers. Studies showed that a high density of Tregs, TAMs, TANs, and PD-L1 in the tumour is associated with higher recurrence rates and poor prognosis in patients with HCC. Conversely, an abundance of CD4 + and CD8 + T cells is associated with significantly better outcomes and fewer cases of recurrence [11, 13, 12, 16, 18, 19]. However, these immune patterns remain underexplored in Danish patients with HCC, who differ from Asian populations where most studies have been conducted. Danish patients generally exhibit a lower prevalence of hepatitis B and C infections, which remains one of the primary aetiologies for HCC, possibly leading to different TME dynamics [2].

This study aims to investigate the association between TME and prognosis (disease-free survival, cancer-specific survival, and overall survival) in Danish HCC patients undergoing liver resection. We hypothesise that a high density of immune-suppressive cells (Tregs, TAMs, and TANs) and PD-L1 in combination with a sparse presence of immune-supportive cells is associated with poor survival outcomes for patients resected for HCC. Furtherly, that these results will enable personalised treatment for this patient group.

## Materials and Methods

We performed an observational cohort study of patients diagnosed with HCC and treated with liver resection between January 2000 and December 2016. We included all patients with available tumour tissue stored in the diagnostic biobank at the Departments of Pathology at Aarhus University Hospital, Denmark, and Copenhagen University Hospital, Rigshospitalet, Denmark. The study was approved by the Central Denmark Region Committees on Health Research Ethics (journal-nr.: 1–10-72–104-17).

The following information was obtained from the patient’s medical records: sex, age at diagnosis, Child-Pugh score, HCC aetiology, microvascular invasion, tumour size, number of tumours, and American Joint Committee on Cancer staging system. None of the patients received systemic treatment prior to surgery.

Resected tumour tissue and corresponding normal liver tissue from all patients were analysed to investigate the density of immune cells in three different areas of the liver: tumour centre, invasive margin, and normal liver tissue. The immune cells of interest were CD8 + T cells, CD4 + T cells (CD4+/FOX-P3-), Tregs (CD4+/FOX-P3+), TANs (CD66b+), and TAMs (CD68+).

Patients determined not representative, patients without available tissue for analysis, patients without complete immunological data, and patients treated for HCC with approaches other than resection were excluded from the study.

### Biomarker Assessment

Formalin-fixed, paraffin-embedded tumour tissue blocks containing areas of the tumour centre, invasive margin, and corresponding normal tissue were selected by a trained pathologist (GLW) and retrieved from the diagnostic biobanks.

Three 3-µm sections were cut from each formalin-fixed, paraffin-embedded tumour tissue block for biomarker assessments. Sections were mounted on SuperFrost Plus slides (ThermoFisher) and incubated at 60 °C for 1 h. Two sections were stained with immunohistochemistry panels targeting the tumour microenvironment and one section was stained with a single stain targeting PD-L1.

### Immunohistochemistry

The immunohistochemistry panels visualising the microenvironment were performed on the Ventana Discovery platform (Roche Diagnostics, North America) with standard settings and reagents for deparaffination, rehydration, heat-induced epitope retrieval, and blocking of endogenous peroxidase activity.

Two panels in total were analysed: one 3-plex panel (CD8 + T cells, CD4 + T cells and Tregs (FOX-P3)) and one dual stain panel (TANs (CD66b+) and TAMs (CD68+)). All panels were an asequential procedure with sequences of primary antibody, secondary antibody, and visualisation (see additional online material: supplementary Table S1 for details) completed with haematoxylin counterstain. Sections of normal tonsil tissue were applied on every slide as a positive control.

The PD-L1 assay was performed on Autostainer Link 48 (Agilent, Santa Clara, United States) with standard settings and reagents recommended for the companion diagnostic assay (see supplementary materials for details). Sections of normal tonsil and placenta were applied on every slide as a positive control. A trained pathologist (TS) evaluated all the stained PD-L1 according to guidelines defining ≥ 1%, ≥ 5%, or ≥ 10% tumour cell expression.

### Digital Imaging and Automated Analysis

The stained tissue sections were scanned at 20x magnification as bright-field whole slides using NanoZoomer HT 2.0 (Hamamatsu Photonics K.K., Hamamatsu City, Japan). Image analysis was performed using Visiopharm Integrator System Version 2020.08.1.8403 (VIS, Visiopharm A/S, Hørsholm, Denmark).

In VIS (Visiopharm A/S), the areas of the tumour centre, invasive margin, and normal tissue were manually outlined on the digital images according to instructions from a trained pathologist (GLW). Areas with necrosis and tissue or technical artefacts were excluded prior to image analysis.

The immune cells were quantified separately in the tumour centre, invasive margin, and normal tissue.

Evaluation of the biomarkers via image analysis was based on a U-net [20] for each IHC panel, which were trained using manually annotated images of 512 × 512 pixels (RGB) in VIS’s Author AI (Visiopharm A/S). Learning rates were based on Adam Optimisation [21] and data augmentation was utilised. All feature maps of U-nets with added mean filters were subsequently classified by thresholding, and postprocessing algorithms (morphological operations and changes by area or surrounding) further enhanced segmentation. Immune cell area-based percentage levels were calculated as the area of the specific cell types relative to the area of the specific region of interest (ROI).

### Statistical Analyses

The density of the individual immune cells of interest in the three tissue compartments was compared using paired Wilcoxon signed-rank test. Differences in cell abundance were analysed for their association with patient prognosis (disease-free survival, cancer-specific survival, and overall survival) through univariable Cox proportional hazard models and reported as hazard ratios (HR) with 95% confidence intervals (95% CI). Overall survival was defined as the time interval between the date of resection and the date of death from any cause, censored at the last follow-up date. Disease-free survival was defined as the interval from the time of resection to the first confirmed recurrence, censored at the last follow-up date. Cancer-specific survival was defined as the interval from the time of resection to the date of death directly caused by cancer, censored at the last follow-up date.

Interactions between immune cells were assessed with Spearman’s Rho.

Unsupervised hierarchical clustering of immune cell densities (Euclidean distance with complete linkage) was used to identify subgroups of samples. Visual inspection of the resulting dendrogram identified 3 distinct subgroups. Subgroup characteristics were identified by post hoc comparisons of immunological and clinical characteristics. Differences between clusters were evaluated using the paired Wilcoxon signed-rank test, and their association with prognosis was analysed with Kaplan-Meier survival curves with log-rank tests.

All analyses were performed with R Statistical Software (v 4.2.2; R Core Team, 2022) and a significance threshold of 0.05.

## Results

### Patient Characteristics

In total, we included 123 patients with HCC, treated with liver resection, and with available tissue stored in the Danish Cancer Biobank. Of these, 48 patients were excluded: 15 patients had necrosis and were determined not representative, 24 patients had no resected tumour and liver tissue available for analysis, and five patients were treated with liver transplantation. The remaining four patients were excluded due to panel analysis failures. Consequently, 75 patients were included in this study.

The median follow-up was 44 months (IQR: 0.69–182 months) and during the entire study period, 51% of the included patients had a recurrence. Overall, 38/48 patients died during the 320 patient-years. Two patients lacked recurrence registration, and their recurrence status is unknown.

Patient demographics at the time of liver resection are shown in Table 1. The majority were male (72%), with a median age of 66 years (IQR: 58–72 years). The majority had no known liver disease at the time of liver resection (51 patients, 68%) and 25 patients (33%) had cirrhosis. The median size of the largest tumour was 50 mm (IQR: 35–85 mm), with most patients having a single tumour (69%) and no microvascular invasion (57%).

**Table 1:** Baseline characteristics

	All patients, *n* = 75
Sex, *n* (%)	
Women	21 (28)
Men	54 (72)
Age, years, median (IQR)	66 (58–72)
Diameter of the largest tumour, mm, median (IQR)	50 (35–85)
Number of tumours, *n* (%)	
1 2 > 2 Missing	52 (69) 7 (9) 1 (1) 15 (20)
Vascular invasion, *n* (%)	
Yes No Missing	13 (17) 43 (57) 19 (25)
Liver disease, *n* (%)	
Alcoholic cirrhosis Hepatitis C virus Hepatitis B virus Non-alcoholic steatohepatitis No known liver disease Other	8 (11) 7 (9) 1 (1) 5 (7) 51 (68) 6 (8)
Cirrhosis, *n* (%)	
Yes No	25 (33) 50 (66)
AJCC stage, *n* (%)	
I II IIIA IIIB IIIC Missing	36 (48) 23 (31) 11 (15) 1 (1) 1 (1) 3 (4)
BCLC, *n* (%)	
Very early stage (0) Early stage (A) Intermediate stage (B) Missing	24 (32) 41 (55) 0 (0) 10 (13)

Baseline characteristics of study population. Categorical variables are presented as frequency, n, with percentages, %, and continuous variables are presented as median with interquartile range, IQR. Abbreviations: AJCC stage: American Joint Committee on Cancer (AJCC) staging system. BCLC: Barcelona Clinic Liver Cancer staging system

### Individual Immune Cell Analysis

**Fig. 1:**
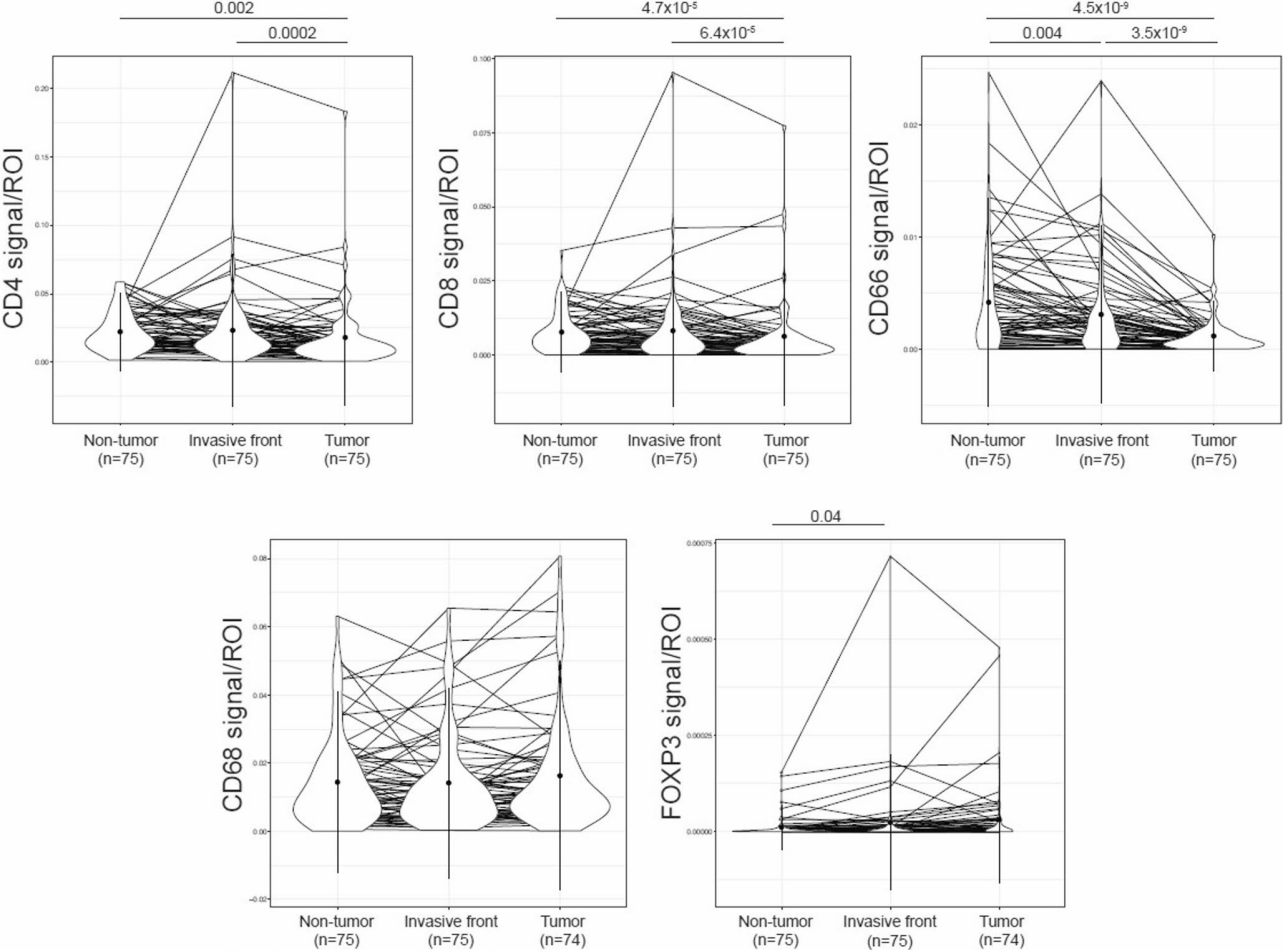
Density of the individual immune cells of interest in the three investigated compartments (tumour, invasive front, and corresponding normal liver tissue) compared with Wilcoxon signed-rank test

The distribution of immune cells in the tumour centre, invasive margin, and normal liver tissue is illustrated in Fig. 1. Among the 75 patients, CD4 + and CD8 + T cells and TANs were less abundant in the tumour centre than in the invasive margin and normal liver tissue, while TAMs and Tregs were more evenly distributed across all compartments.

A higher density of CD4 + T cells in the invasive margin was significantly associated with reduced overall survival (HR = 1.43, 95% CI: 1.13–1.82, *p* = 0.003). Conversely, a higher density of TAMs in the tumour centre was associated with improved cancer-specific survival (HR = 0.53, 95% CI: 0.28–1.00, *p* = 0.05) (Table 2).

**Table 2:**
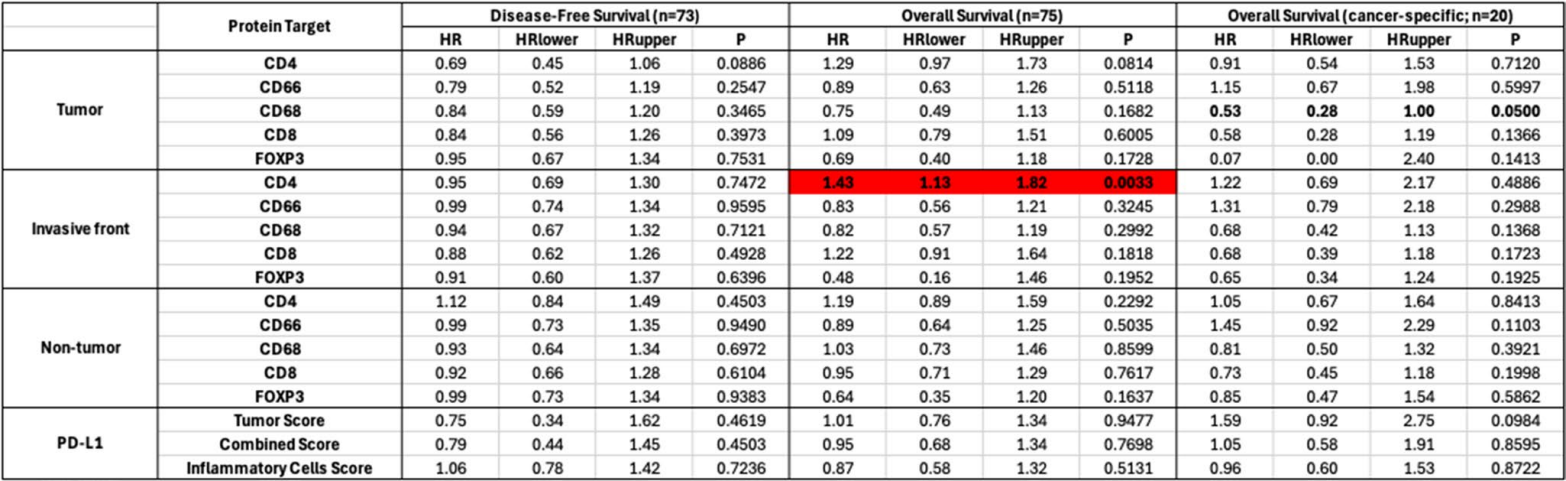
Univariable Cox proportional hazards regression analysis

### Interactional Relationship and Cluster Analysis

**Fig. 2:**
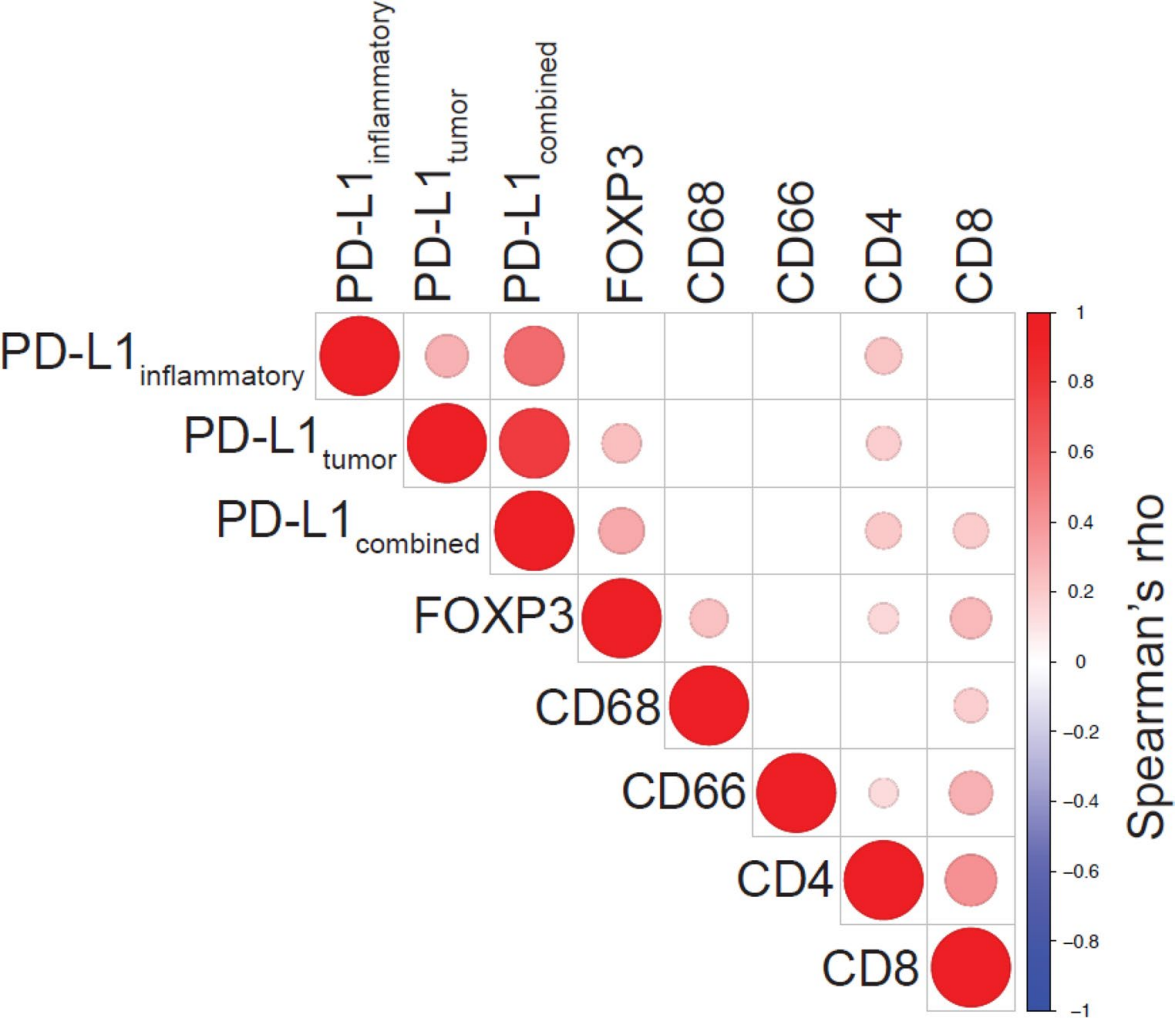
Interactional relationship between the immune cells of interest and PD-L1 investigated with Spearman’s rho correlation coefficients. The strength of the monotonic relationship between two variables is depicted by the size and saturation of the circles and the y-axis

Spearman’s Rho analysis indicated a positive correlation between PD-L1 and both CD4 + and CD8 + T cells, suggesting that higher PD-L1 levels correlate with increased CD4 + and CD8 + T cell densities. Additionally, a negative correlation was observed between CD4 + and CD8 + T cells. Results are presented in Fig. 2.

**Fig. 3:**
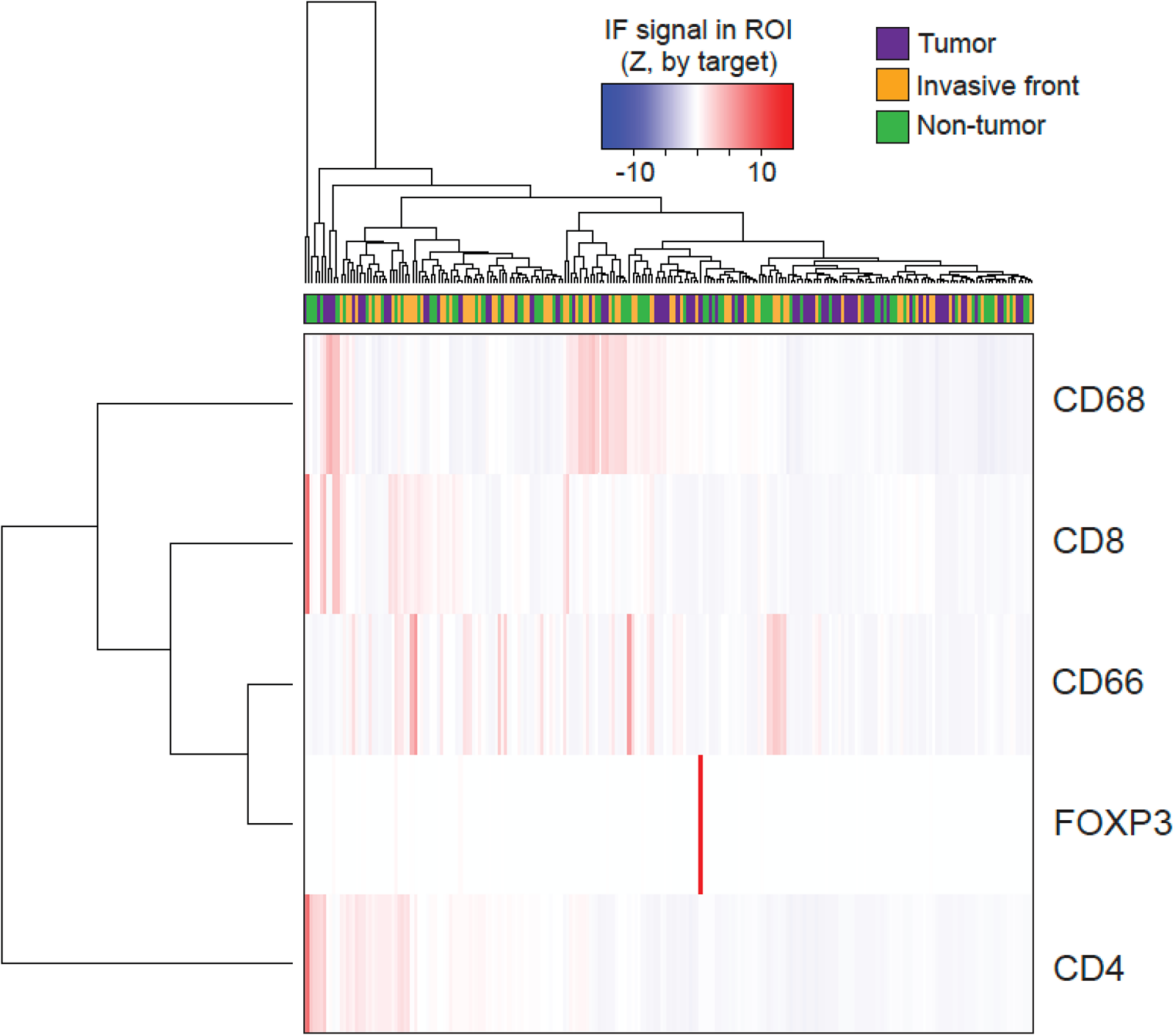
Identification of patient clusters based on immune cell densities in samples from the three investigated compartments (tumour, invasive front, and corresponding normal liver tissue) with hierarchical clustering

**Fig. 4:**
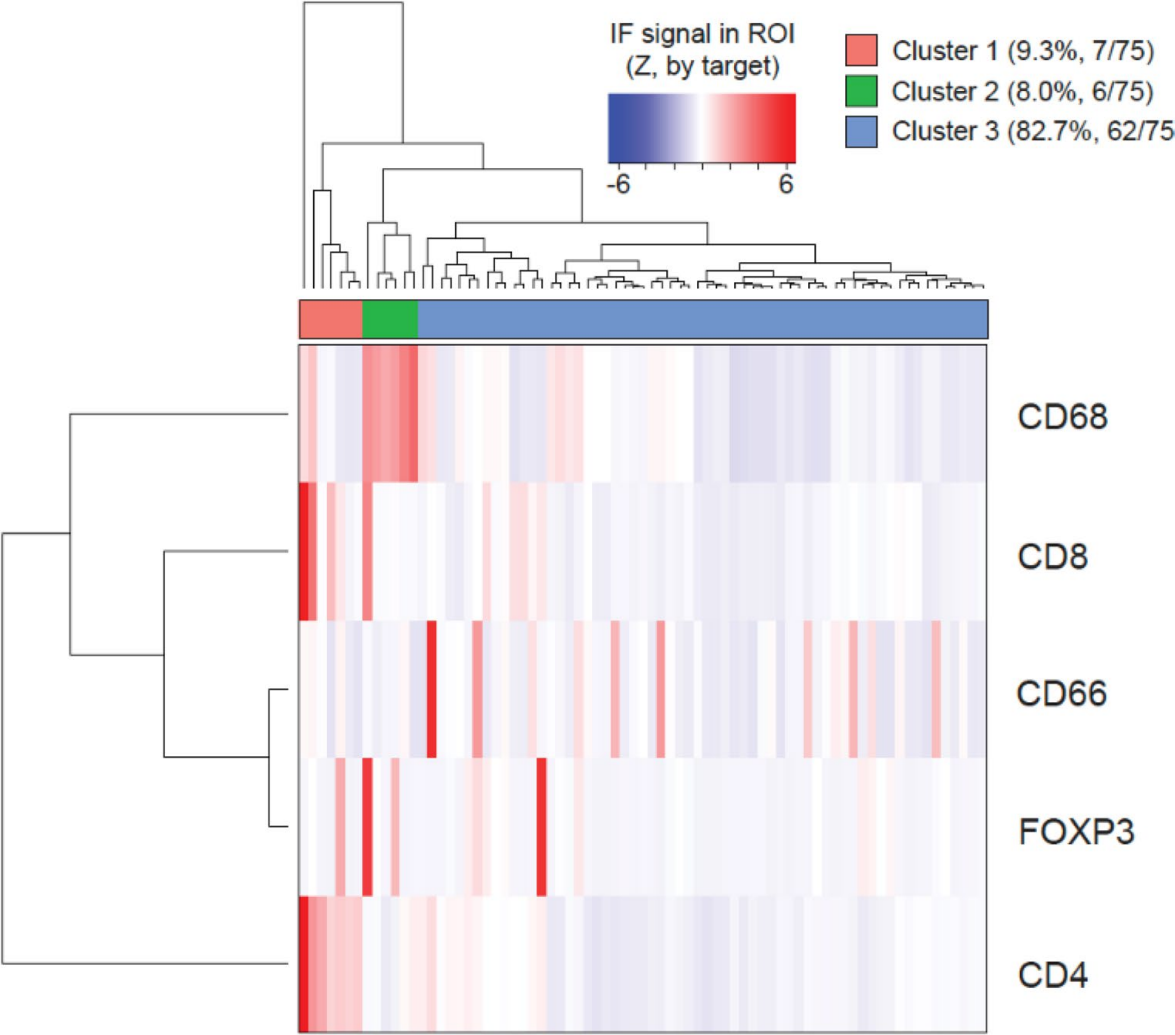
Identification of the three immune clusters among tumour samples with hierarchical clustering

**Fig. 5:**
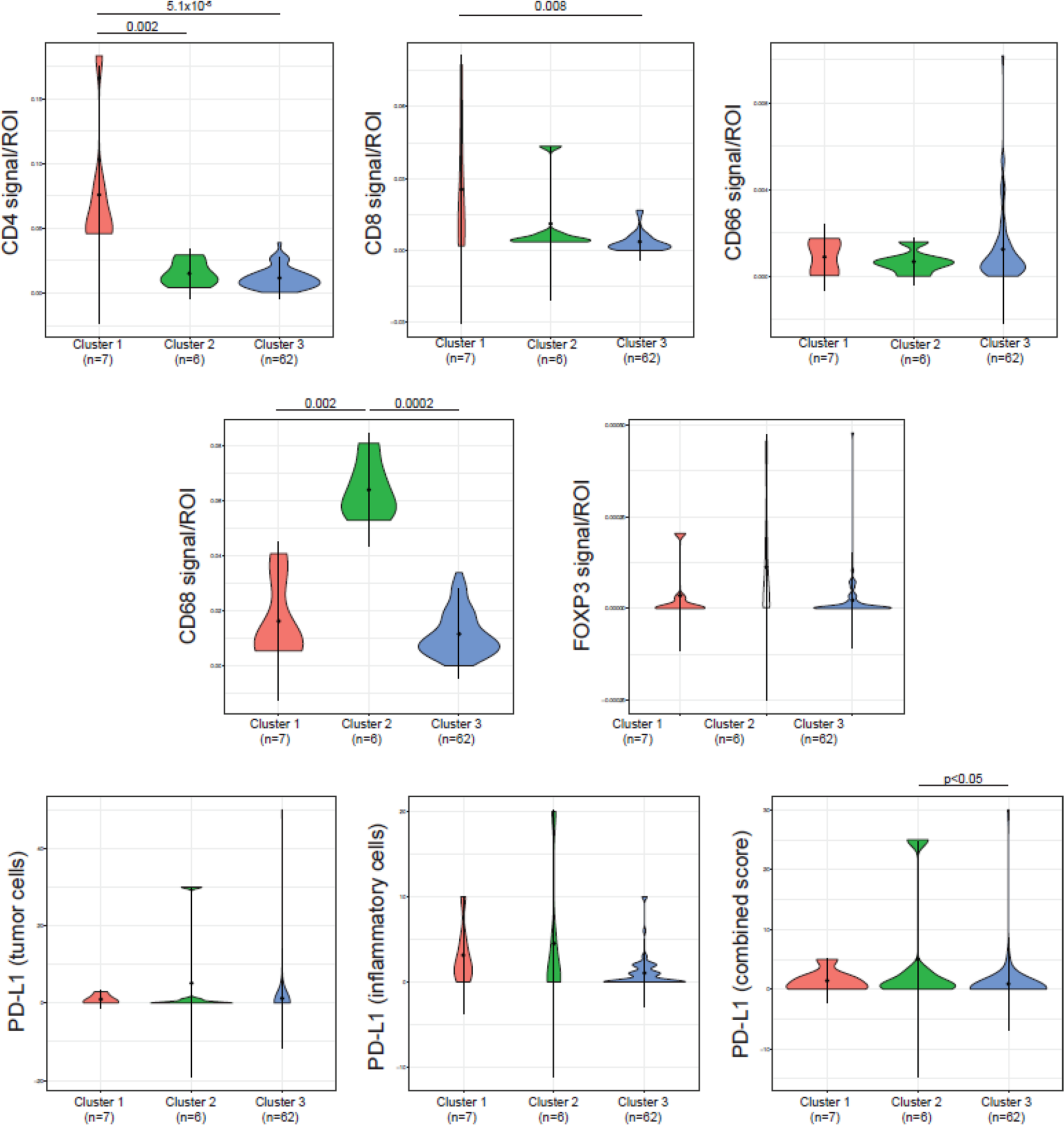
Differences in density of immune cells and PD-L1 among the three clusters compared with Wilcoxon signed-rank test

Hierarchical clustering categorised patients into three groups based on tumour tissue characterisation: cluster 1 (*n* = 7) with high densities of CD4 + and CD8 + T cells, cluster 2 (*n* = 6) with a high density of TAMs, and cluster 3 (*n* = 62) with low immune cell densities overall (Figs. 3 and 4). A Wilcoxon signed-rank test confirmed significant cluster differences (Fig. 5).

**Fig. 6:**
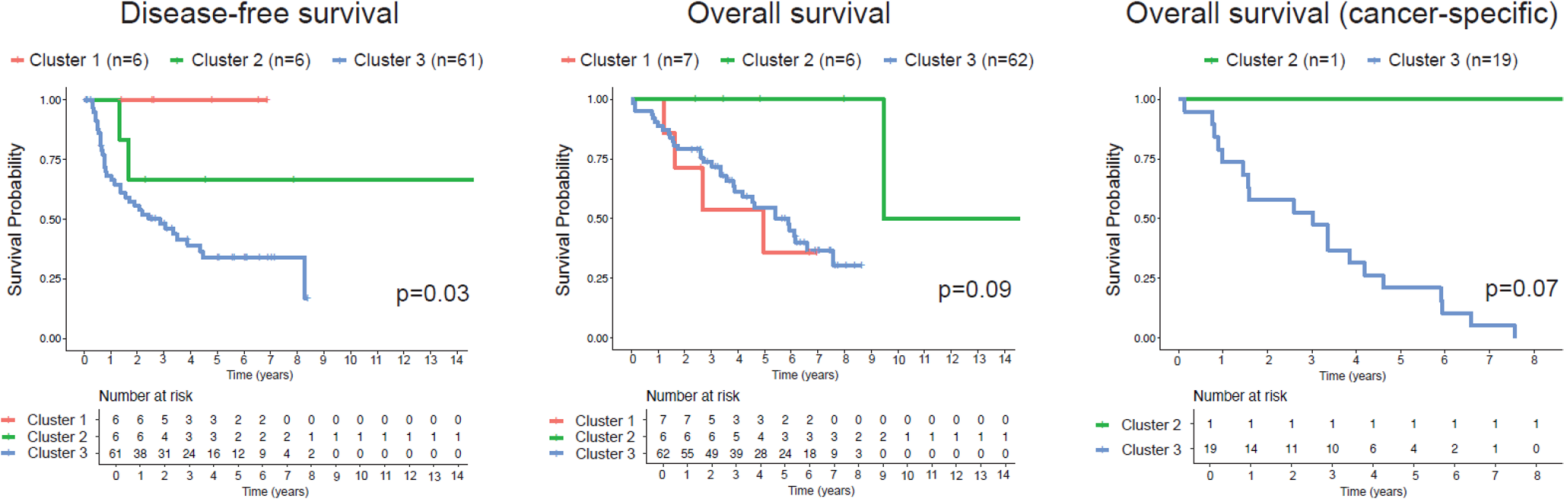
Kaplan-Meier curves with Log-rank p-values. Disease-free survival: median survival time for cluster 1: 951 days, cluster 2: 1245.5 days, and cluster 3: 725.5 days. Overall survival: median survival time for cluster 1: 974 days, cluster 2: 2333 days, and cluster 3: 1358 days

Cluster 3 (immune-low cluster) exhibited significantly worse disease-free survival than clusters 1 and 2 (*p* = 0.03). No significant differences in overall or cancer-specific survival were observed across clusters (Fig. 6).

**Fig. 7:**
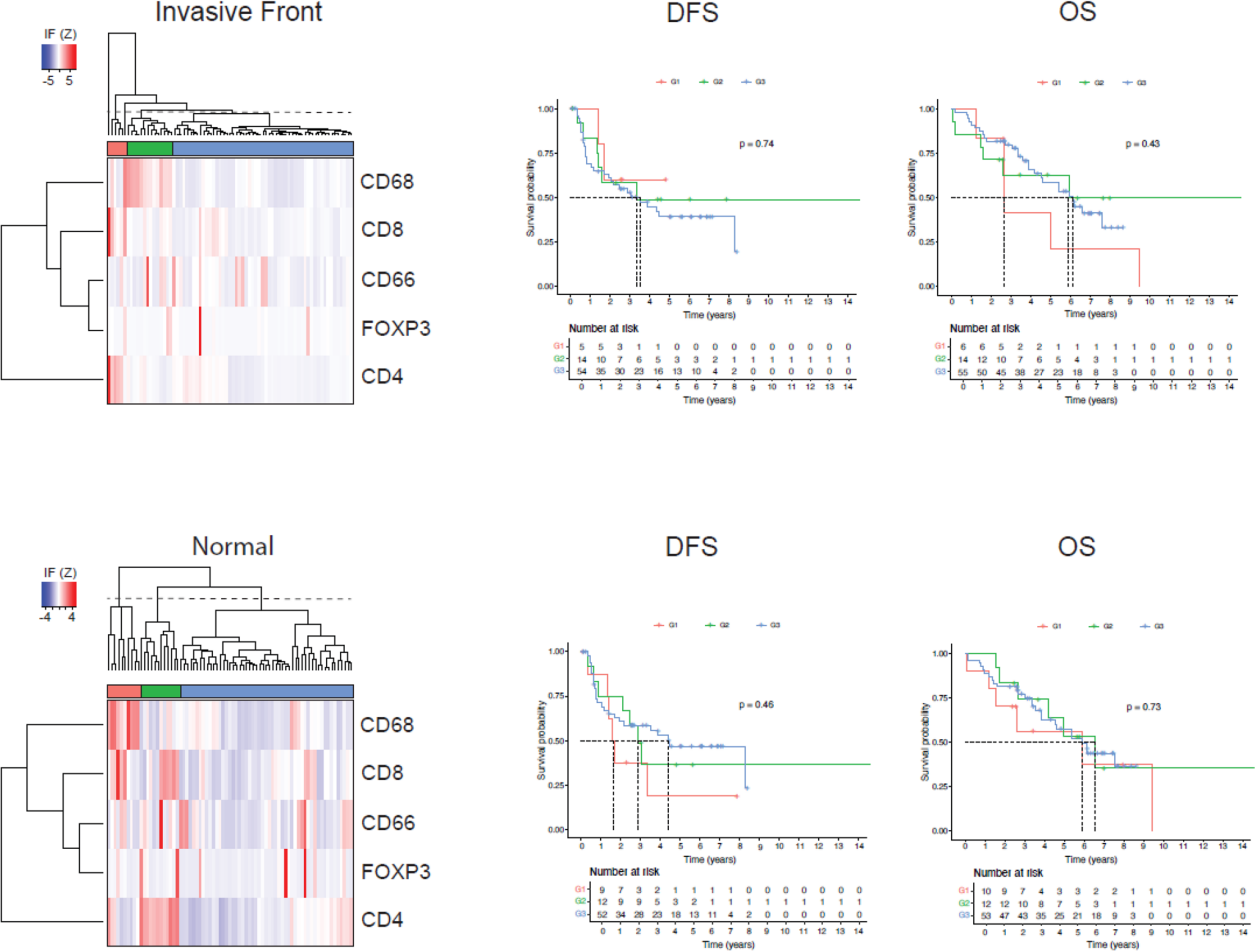
Identification of the three immune clusters among samples from the invasive front and corresponding normal liver tissue with hierarchical clustering and corresponding Kaplan-Meier survival curves

A similar hierarchical clustering of patient samples from the invasive margin and normal liver tissue showed no correlation to survival (Fig. 7).

## Discussion

This study analysed immune cell distributions in tissue samples from 75 patients with HCC treated with liver resection. The main findings were that CD4 + and CD8 + T cells were less abundant in the tumour centre compared to the invasive margin and normal liver tissue, while TAMs and Tregs were evenly distributed across all compartments. Higher CD4 + T cell density in the invasive margin was significantly associated with decreased overall survival, and a high TAM density in the tumour centre was significantly associated with improved cancer-specific survival. Additionally, we found that patients with a low density of all immune cells had a significantly higher risk of recurrence.

The reduced density of CD4 + and CD8 + T cells in the tumour centre aligns with previous findings that CD4 + T cell levels decrease as HCC progresses, impairing CD8 + T cell activation [22]. This diminished activation compromises the anti-tumour immune response, contributing to poorer overall survival and higher recurrence rates. Such findings reinforce the protective role of these immune cells in controlling tumour progression [11, 22].

In contrast to our findings, other research has consistently found that high densities of CD4 + and CD8 + T cells in HCC have been associated with improved survival and reduced recurrence in patients treated with liver resection [23–26]. However, most of these studies assessed immune infiltration within broad regions, tumour tissue and adjacent non-tumour liver tissue, without distinguishing between specific compartments. The lack of compartment-specific analysis may obscure spatially distinct immune patterns with distinct biological and prognostic implications. By differentiating immune cell densities across multiple tissue compartments, our study suggests that the prognostic impact of CD4 + and CD8 + T cells may depend on their spatial localisation within the tumour microenvironment. Importantly, most prior studies were conducted in Asian cohorts with a high prevalence of chronic viral hepatitis, which may shape immune activation and tumour–host interactions differently than in Northern European populations. In contrast, the present cohort included predominantly patients without clinically evident chronic disease. Differences in the underlying inflammatory environment may therefore contribute to the divergent associations observed. Together, these findings underscore the context-dependent nature of immune–prognostic relationships in HCC.

While higher densities of Tregs and TAMs were not directly correlated with poor survival or recurrence, their known association with poor prognosis and recurrence remains relevant [11]. TAMs recruit Tregs, indirectly facilitating their immunosuppressive role, and are associated with the promotion of angiogenesis, cancer cell proliferation, invasion, and metastasis. This pro-tumour activity is especially pronounced when the TAM population is polarised towards the M2 subtype, which in themselves exhibit immune suppressive functions and are generally associated with poorer survival outcomes. Unlike the M2 subtype, the M1 macrophages, which promote the elimination and degradation of tumour cells through their pro-inflammatory functions, are usually associated with improved survival [27]. The association between a high TAM density in the tumour centre and better cancer-specific survival, observed in our study, contrasts with previous reports linking TAMs to poorer outcomes and increased recurrence rates [27]. Typically, the majority of macrophages in the TME consists of the M2 subtype, however, we cannot be certain that this is the case in the study we have conducted, since we were not able to obtain a marker to differentiate between subtypes, which in turn could explain the discrepancy between our findings and previous ones.

Our study was the first clinical study demonstrating the association of the immune microenvironment in HCC with prognosis in a Danish cohort of surgical patients undergoing liver resection for HCC. However, it is important to keep in mind that the TME is a complex and dynamic system. This study focused on selected immune cell populations as one defined dimension of that system, rather than providing a comprehensive characterisation. Our findings should therefore be interpreted within this context.

The population of this study differs from the majority of the literature due to a low prevalence of cirrhosis and chronic viral hepatitis. However, as this study exclusively includes patients with preserved liver function undergoing liver resection, the majority (66%) with non-cirrhotic HCC, it represents a clinically selected subgroup of early-stage HCC rather than the full population-based spectrum of the disease. A further strength lies in the study’s homogenous patient cohort with correction for important confounding factors. However, the study’s sample size of 75 patients is a limitation, potentially reducing the power to detect clinically significant trends.

The relationship between tumour-infiltrating lymphocytes, other immune cells, and HCC prognosis has yet to be integrated into clinical decision-making. Preoperative evaluations of the tumour microenvironment (TME) could personalise HCC treatment and improve outcomes following resection. However, a reliable, non-invasive method for TME assessment is urgently needed. While liver biopsies offer one approach, they carry risks such as bleeding and tumour seeding, underscoring the necessity of developing safer techniques [28]. Future studies should prioritise non-invasive or minimally invasive tools for immune profiling to facilitate their incorporation into clinical practice.

Given the exploratory nature of this study and the limited sample size, formal power calculations were not applicable. Consequently, the identified immune cell subgroups as well as the other findings of this study require further validation in larger, multi-centre studies within similar populations, such as Danish or other Western cohorts, to validate these findings and ensure their applicability to different patient demographics. Such studies could help refine the understanding of regional differences in the TME and how they influence HCC prognosis. Additionally, prospective research should focus on assessing the TME as a prognostic biomarker. Validation of immune cell profiling through larger cohorts and longer follow-ups could establish its utility in guiding clinical decision-making. If these biomarkers are consistently linked to survival outcomes, they could play a pivotal role in stratifying patients for personalised treatment strategies, particularly in selecting candidates for liver resection or adjuvant therapies.

In conclusion, this study investigated the association between the prognostic value of the TME and the survival outcome of patients with HCC undergoing liver resection. Specifically, a high density of CD4 + T cells in the invasive margin was significantly associated with a higher risk of mortality, while a high density of TAMs in the tumour centre was significantly associated with a lower risk of cancer-related mortality. Cluster analysis revealed that patients with low densities of all immune cells had significantly worse disease-free survival compared to other patients. These findings emphasise the importance of immune profiling in HCC prognosis and suggest that, after validation in prospective studies, these insights could contribute to personalised treatment strategies for HCC patients.

## Supplementary Information

Below is the link to the electronic supplementary material.

Supplementary Material 1 (DOCX 24.0 KB)

## Data Availability

The Danish Data Protection Agency does not allow open access to the data included in this study. However, reasonable requests for additional analyses on the dataset can be made to the corresponding author.

## Declarations

### Declarations of Generative AI and AI-assisted Technologies in the Writing Process

During the preparation of this work, the authors used ChatGPT from OpenAI to improve clarity, grammar and readability of the manuscript. After using this tool/service, the authors reviewed and edited the content as needed and take full responsibility for the content of the publication.
