# Supplementary material for: Survival and Tumour Microenvironment in Spatially Distinct Regions in Patients Resected for Hepatocellular Carcinoma: A Multicentre Study
Source: J Gastrointest Cancer. 2026 Apr 15;57(1):91. doi: 10.1007/s12029-026-01467-1 (PMC13083490; doi:10.1007/s12029-026-01467-1)
Supplement: Supplementary file 1 — Supplementary Material 1 (DOCX 24.0 KB) [file 12029_2026_1467_MOESM1_ESM.docx]

*Survival and tumour microenvironment in spatially distinct regions in patients resected for hepatocellular carcinoma: a multicentre study*

Sophie Bull Nordkild^1-3^, Jeanett Klubien^1-3^, Delal Akdag^1-3^, Colm O’Rourke^4^, Jeanette Bæhr Georgsen^5,6^, Patricia Switten Nielsen^5,6^, Torben Steiniche^5,6^, Gerda Elisabeth Villadsen^6,7^, Anders Riegels Knudsen ^6,8^, Gro Linno Willemoe^9^, Jesper Bøje Andersen^4^, Susanne Dam Nielsen^1,3,10^, Hans-Christian Pommergaard^1,2,10^

^1^Department of Digestive Diseases, Transplantation and General Surgery, Rigshospitalet, Copenhagen University Hospital, Copenhagen, Denmark

^2^Hepatic Malignancy Surgical Research Unit (HEPSURU), Department of Digestive Diseases, Transplantation and General Surgery, Copenhagen University Hospital, Copenhagen, Denmark

^3^Viro-immunology Research Unit, Department of Infectious Diseases, Rigshospitalet, Copenhagen University Hospital, Copenhagen, Denmark

^4^Biotech Research and Innovation Centre (BRIC), Department of Health and Medical Sciences, University of Copenhagen, Copenhagen, Denmark

^5^Department of Pathology, Aarhus University Hospital, Aarhus, Denmark

^6^Department of Clinical Medicine, Aarhus University, Aarhus, Denmark

^7^Department of Hepatology and Gastroenterology, Aarhus University Hospital, Aarhus, Denmark

^8^Department of Surgery, Aarhus University Hospital, Aarhus, Denmark

^9^Department of Pathology, Rigshospitalet, Copenhagen University Hospital, Copenhagen, Denmark

^10^Institute for Clinical Medicine, Panum Institute, University of Copenhagen, Copenhagen, Denmark

**Corresponding author’s email**

Email: [hans-christian.pommergaard@regionh.dk](mailto:hans-christian.pommergaard@regionh.dk)

**Journal name**: Journal of Gastrointestinal Cancer

**Supplementary material**

Table S1. Multiplexing immunohistochemistry panels.

|  | **Clone** | **Vendor** | **Conc** | **Inc.** | **Secondary ab** | **Detection** |
| --- | --- | --- | --- | --- | --- | --- |
| **Panel 1** | | | | | | |
| CD8 | C8/144B | Agilent | 1:150 |  | OmniMap ms hrp | Chromomap DAB |
| CD4 | SP35 | Ventana | RTU |  | OmniMap rb hrp | Discovery Purple |
| Fox-P3 | EPR22102-37 | Abcam | 1:10 |  | UltraMap rb alk phos | Discovery Yellow AP |
| **Panel 2** | | | | | | |
| CD66b | G10F5 | BD Bioscience | 1:200 |  | OmniMap ms hrp | Chromomap DAB |
| CD68 |  |  |  |  | OmniMap ms hrp | Discovery Purple |
| **Panel 3** | | | | | | |
| PD-L1 | 28-8 | Agilent | RTU |  | Linker, anti-rabbit | DAB+ |
